# Association between migration paths and mental health of new-generation migrants in China: The mediating effect of social integration

**DOI:** 10.3389/fpsyt.2022.967291

**Published:** 2022-09-07

**Authors:** Fenfen Zhou, Boli Peng, Muyang Chu, Hui Zhang, Lishuo Shi, Li Ling

**Affiliations:** ^1^Department of Medical Statistics, School of Public Health, Sun Yat-sen University, Guangzhou, China; ^2^Sun Yat-sen Center for Migrant Health Policy, Sun Yat-sen University, Guangzhou, China; ^3^Department of Actuarial Science, School of Insurance, Guangdong University of Finance, Guangzhou, China; ^4^Department of Health Policy and Management, School of Public Health, Sun Yat-sen University, Guangzhou, China; ^5^The Sixth Affiliated Hospital, Sun Yat-sen University, Guangzhou, China

**Keywords:** mental health, new-generation migrants, migration paths, social integration, urban, rural, China

## Abstract

**Background:**

The new-generation migrants born in 1980 and later are large and vulnerable internal migrants in China. Migration paths and social integration are important factors to explain for their mental health. However, they faced difficulties in social integration varying from migration paths. We aimed to explore the mediating role of social integration between migration paths and the mental health of new-generation migrants.

**Methods:**

The migration paths included urban-to-urban, urban-to-rural, rural-to-urban and rural-to-rural. Mental health was assessed by the Kessler Screening Scale for Psychological Distress (K6) and the Perceived Stress Scales (PSS-4). Social integration was measured by economic integration, life integration, maintenance of the local culture, acceptance of the host culture and psychological integration. Multiple linear regressions with bootstrapping were used to examine the mediating effect.

**Results:**

A total of 9,830 new-generation migrants were included in this study. The mean age was 26.92 (*SD* = 4.47) years and the proportion of rural-to-urban migrants was 63.7%. Compared with the new generation of rural-to-rural migrants, rural-to-urban migrants had higher psychological distress (β = 0.305, 95% CI: 0.152–0.458) and perceived stress (β = 0.328, 95% CI: 0.199–0.456). The bootstrapping test found that two dimensions (“life integration” and “acceptance of the host culture”) of social integration as a mediator weakened the negative effect of the rural-to-urban migration path on the mental health of new-generation migrants.

**Conclusion:**

Rural-to-urban migrants had poorer mental health, and the association was mediated by their poorer social integration. The migration policies developed to enhance social integration could effectively improve the mental health of new-generation migrants.

## Introduction

By the end of 2018, there were 241 million internal migrants (IMs) in China, accounting for 17.3% of the overall population according to China Statistical Yearbook 2019 (1). The emergence of IMs in China could be linked to the Hukou system. Since the 1980s, because of the high demand for labor in urban areas, the Chinese government has loosened its control over Hukou management, and allowed the rural Hukou population to work and do business in urban areas (2, 3). In China, this migrant population is defined as IMs. The definition of the generation of IMs was based on the date of birth. According to the definition of Chinese National Bureau of Statistics, those born in January 1980 and later were classified as the “new generation,” while people born before 1980 were classified as the “old generation” (4, 5). The year 1980 was chosen because it was the year that China began comprehensive social and economic reforms (6, 7). Based on the Report on Chinese Migrant Population Development in 2018, the population size of new-generation migrants who were born in 1980 and later continuously increases rapidly, accounting for 65.1% of the IMs (8). Compared with the old-generation migrants, new-generation migrants have higher professional ambitions and higher urban lifestyle preferences (9).

Social integration is a multi-dimension concept used to explain the behavior, adaptation, acculturation process and self-identity of migrants (10), without clear and unified definition among transdisciplinarity (11). Some studies use multi-dimensional indexes, covering cultural, economic and psychological dimensions, while others use one-dimensional indicators of economic conditions. Multi-dimensional indicators may deviate from the overall evaluation of social integration of migrants; one-dimensional indicators often ignore the complexity of social integration. For example, Wu chose an urban household registration index as a measure of social integration (12). The theory of social integration was developed to understand the behavior, adaptation, cultural integration, and acculturation processes of migrants, although most have been applied in Western societies (10, 13). Thus, the measurement network of social integration is uncertain, and the typical operational definition is a scale based on individual summary scores of different types of social roles or social networks (14). However, social integration was not only related to individual characteristics but also the characteristics of “the place of origin” and “the host cities” (15).

Evidence from previous studies suggested that during the process of migration, the new-generation migrants were also confronted with social integration challenges (16). The greater the changes in the socio-economic environment resulting from the migration paths (i.e., migration from the place of origin to the host city), the greater the social integration difficulties faced by the new-generation migrants (17). Migration paths were reported to be important predictive factors of common mental disorders among migrants across the world (18). Furthermore, with migration paths classified as urban-to-urban, urban-to-rural, rural-to-urban and rural-to-rural, previous studies found that rural-to-urban migrants had higher psychological distress among the IMs (19). The stress from social integration might negatively affect the mental health of new-generation migrants (20–23), which may endanger their family relationships and even pose a serious threat to the economic development of the place of origin and the host city (2, 24–26).

The new-generation migrants are high-risk groups facing mental health problems. Migration paths and social integration have been identified as the important factors affecting the mental health of migrants. However, there is little research on the relationship between social integration and mental health based on different migration paths, especially for the new-generation migrants. The present study was aimed to explore the mediating effect of social integration on the relationship between migration paths and mental health among the new-generation migrants by using the secondary public database of the National Internal Migrant Dynamic Monitoring Survey in 2014, which would help identify the vulnerable groups and provide more effective interventions according to the mediator.

There are two hypotheses in this study: (1) Migration paths are associated with mental health. (2) After the adjustment of social integration, the above correlation might be weakened, and it is speculated that migration paths could affect the mental health among new-generation migrants mediated by social integration.

## Materials and methods

### Study design and participants

The data was extracted from the National Internal Migrant Dynamic Monitoring Survey in 2014. This survey was a cross-sectional survey conducted by the National Population and Family Planning Commission of China in May 2014, which included a thematic survey on the social integration and health status of internal migrants (IMs). Because NIMDMS changed the survey theme every year, the data in 2014 was the most comprehensive thematic survey on the social integration of China’s migrant population (27). The thematic survey was carried out in eight “demonstration pilot cities” with different degrees of migration and social integration programs, including Beijing (the capital of China), Zhengzhou and Chengdu (two provincial capitals), Xiamen, Jiaxing, Qingdao, Shenzhen and Zhongshan (five economic centers in eastern and southern coastal regions), which throughout the eastern, central, and western areas of China.

A stratified multi-stage probability proportional to size (PPS) sampling method was used to sample the respondents from eight “demonstration pilot cities.” First, taking the annual report data in 2013 as the basic sampling frame, the sub-districts or townships within each district or county were selected. Second, depending on the size of the IMs, neighborhoods or villages were drawn from the selected sub-districts or townships. Finally, in each neighborhood or village, 20 respondents between the ages of 15 and 59 who had lived locally for at least 1 month were selected. A total of 15,999 respondents were included. A face-to-face interview questionnaire survey was conducted among the respondents. Interviewers from eight “demonstration pilot cities” have received standardized training from the National Population and Family Planning Commission. At the same time, quality control was implemented in the process of data collection (28).

After removing 12 IMs with incomplete questionnaire information, 2,325 IMs who had not lived in “demonstration pilot cities” for 6 months and 3,832 IMs born before January 1980, there were 9,830 new-generation migrants who were born in January 1980 or later in our study (4).

### Assessment of mental health

Mental health was assessed by psychological distress and perceived stress over the past 30 days. Psychological distress was measured by the 6-item Kessler Scale (K6) (29). The values for each item range from 0 (none of the time) to 4 (all of the time). On a scale of 0 to 24, higher scores indicate worse mental health. It has been proved that K6 has good psychometric properties in the Chinese population (Cronbach’s α = 0.84) (30).

Perceived stress was measured with the 4-item Perceived Stress Scales (PSS-4), which is a brief and effective stress scale for different groups of people (16). PSS-4 values range from 0 to 16, and the higher the score, the higher the perceived stress level. Previous studies have confirmed the satisfactory psychometric properties of the Chinese version of PSS-4 (Cronbach’s α = 0.67) (31).

### Assessment of migration paths and social integration

According to the participants’ Hukou system (registered permanent residence located in a county or a district) and current residence (a county or a district) of the respondents, migration paths were divided into four groups, including rural-to-rural, urban-to-rural, ur-ban-to-rural and rural-to-urban (32).

In this study, social integration consisted of five dimensions and was measured using 27 items (33), referred to Yang’s theoretical framework for social integration (34). There was economic integration (five items), life integration (four items), maintenance of original culture (four items), acceptance of the host culture (four items) and psychological integration (ten items). Reliability analyses found strong internal consistency in 27 items (Cronbach’s α = 0.70). Questions about the dimensions of social integration and the results of reliability analyses were summarized in Supplementary Table 1. Factor analysis was used to calculate the scores of each dimension, and these scores were further converted into a range of 0–100 by the min-max normalization and percentage method. The values of social integration ranged from 0 to 100 for each dimension, with a higher score indicating a higher level of social integration.

### Potential confounding variables

A series of demographics related to mental health reported in previous studies were adjusted in the current study, including age, sex, marital status, nationality, education level, occupation, and Hukou status (35, 36). Due to the various obstacles migrants encountered in the process of migration and the post-migration period, migration characteristics have become the cause of mental illnesses (37). Migration characteristics included the scope of migration, length of residence in the host cities, reasons for migrating and whether people migrate with their families (38). Two other variables were also included in the study, establishing health records and receiving health education, which might be associated with the social integration and mental health of the IMs (39). The variables of establishing health records and receiving health education were both measured by the question contained in the NIMDMS.

### Statistical analysis

Descriptive analysis was conducted for all variables. The continuous variables were expressed in mean (SD) or median (IQR). The categorical variables were reported by numbers and percentages. Analysis of variance, rank-sum test and chi-square test were used to compare the differences of factors in the four migration paths.

Before the establishment of the multivariable linear regression model, bivariate linear regression analysis was used to examine the relationship between mental health and migration paths. In bivariate analysis, considering that the relationship between independent and dependent variables may be masked by potential confounders, in order to avoid overly strict criteria leading to the omission of some meaningful variables, *P* < 0.1 was therefore used as the selection criterion (40). Migration paths, social integration, statistically significant confounding factors (*P* < 0.1) and theoretically proven influencing factors were included in the multivariable linear regression model.

The −2 log likelihood ratio (−2 LLR) test was used to determine whether the mediating effect improved the goodness-of-fit of the model (41). The −2 LLR decreases by 3.84 or more (Chi-square limit of 1 degree of freedom is 0.05), indicating that one model is superior to the other. The mediator was examined by using mediation analysis with PROCESS macro, which was based on the path analytic framework of ordinary least square (OLS) regression (42). The multiple linear regression model 1(not including the mediator) and model 2 (including the mediator) were established, and the coefficients of regression and its 95% confidence interval (CI) were calculated by the bootstrapping method (43). If the value of the mediating effect was greater than zero and the bootstrapping 95% CI did not contain zero, the mediating effect would play a role. Conversely, if the value of the mediating effect was less than zero, it could only be explained by the “suppressing effects” (44). All statistical analyses were conducted using IBM SPSS 26.0.

## Results

### Characteristics of the study sample

The demographic characteristics and migration characteristics of the 9,830 new-generation migrants were presented in Table 1. Overall, the mean age of new-generation mi-grants was 26.92 (*SD* = 4.47) years old, 53.4% of new-generation migrants were male and 59.9% of them were married. Nearly half of them had a secondary school of education level. 86.5% of the respondents had a rural Hukou. More respondents migrated across provinces, and 48.3% of respondents have lived in the host city for 1–5 years. There were 94.4% and 64.1% of respondents migrated for business and migrated with their families, respectively. The new-generation migrants were divided into four migration paths. Most of them migrated from rural to urban (63.7%), followed by rural-to-rural (23.8%), ur-ban-to-urban (11.2%) and urban-to-rural (1.3%), respectively.

**TABLE 1:** Characteristics of 9,830 new-generation migrants from Internal Migrant Dynamic Monitoring Survey in China, 2014.

Variables		Migration paths				*P*-value^a^
	Total (*N* = 9,830) *N*(%)/*M* ± *SD*	Rural-to-Rural (*N* = 2,339) *N*(%)/*M* ± *SD*	Urban-to-Urban (*N* = 1,101) *N*(%)/*M* ± *SD*	Urban-to-Rural (*N* = 129) *N*(%)/*M* ± *SD*	Rural-to-Urban (*N* = 6,261) *N*(%)/*M* ± *SD*	
Age (years)	26.92 ± 4.47	26.76 ± 4.54	28.34 ± 3.91	28.05 ± 3.90	26.72 ± 4.51	<0.001
**Sex**						0.944
Female	4577 (46.6)	1099 (47.0)	513 (46.6)	62 (48.1)	2903 (46.4)	
Male	5253 (53.4)	1240 (53.0)	588 (53.4)	67 (51.9)	3358 (53.6)	
**Marital status**						<0.001
Single	3943 (40.1)	821 (35.1)	420 (38.1)	42 (32.6)	2660 (42.5)	
Married	5887 (59.9)	1518 (64.9)	681 (61.9)	87 (67.4)	3601 (57.5)	
**Nationality**						<0.001
Han	9480 (96.4)	2221 (95.0)	1063 (96.5)	127 (98.4)	6069 (96.9)	
Other	350 (3.6)	118 (5.0)	38 (3.5)	2 (1.6)	192 (3.1)	
**Education level**						<0.001
Primary school and less	307 (3.1)	123 (5.3)	9 (0.8)	1 (0.8)	174 (2.8)	
Secondary school	4685 (47.7)	1462 (62.5)	118 (10.7)	29 (22.5)	3076 (49.1)	
High school	2889 (29.4)	571 (24.4)	268 (24.3)	44 (34.1)	2006 (32.0)	
College and above	1949 (19.8)	183 (7.8)	706 (64.1)	55 (42.6)	1005 (16.1)	
**Hukou**						<0.001
Rural	8501 (86.5)	2324 (99.4)	0 (0.0)	0 (0.0)	6177 (98.7)	
Urban	1329 (13.5)	15 (0.6)	1101 (100.0)	129 (100.0)	84 (1.3)	
**Range of migration**						<0.001
Across the province	5312 (54.0)	1714 (73.3)	683 (62.0)	93 (72.1)	2822 (45.1)	
Within the province	4518 (46.0)	625 (26.7)	418 (38.0)	36 (27.9)	3439 (54.9)	
**Duration of residence in the host city (years)**						<0.001
<1	3074 (31.3)	750 (32.1)	269 (24.4)	31 (24.0)	2024 (32.3)	
1 ∼ 5	4745 (48.3)	1143 (48.9)	554 (50.3)	64 (49.6)	2984 (47.7)	
5 ∼ 10	1521 (15.5)	336 (14.4)	208 (18.9)	28 (21.7)	949 (15.2)	
≥10	490 (5.0)	110 (4.7)	70 (6.4)	6 (4.7)	304 (4.9)	
**Reasons for migration**						0.168
Business	9276 (94.4)	2205 (94.3)	1042 (94.6)	116 (89.9)	5913 (94.4)	
Others	554 (5.6)	134 (5.7)	59 (5.4)	13 (10.1)	348 (5.6)	
**Migrating with families**						<0.001
Yes	6300 (64.1)	1609 (68.8)	715 (64.9)	90 (69.8)	3886 (62.1)	
No	3530 (35.9)	730 (31.2)	386 (35.1)	39 (30.2)	2375 (37.9)	
**Establishment of health records**						<0.001
Yes	2266 (23.1)	318 (13.6)	278 (25.2)	16 (12.4)	1654 (26.4)	
No	7563 (76.9)	2021 (86.4)	823 (74.8)	113 (87.6)	4606 (73.6)	
**Receiving health education**						<0.001
Yes	6604 (67.2)	1319 (56.4)	809 (73.5)	78 (60.5)	4398 (70.2)	
No	3226 (32.8)	1020 (43.6)	292 (26.5)	51 (39.5)	1863 (29.8)	
**Social integration**						
Economic integration	44.68 ± 11.51	44.04 ± 10.52	48.65 ± 13.13	47.33 ± 11.63	44.16 ± 11.41	<0.001
Life integration	20.75 ± 15.70	15.41 ± 11.58	29.16 ± 17.83	19.90 ± 15.01	21.28 ± 15.91	<0.001
Maintenance of original culture	65.45 ± 16.53	67.03 ± 16.18	61.31 ± 16.88	65.82 ± 18.61	65.59 ± 16.43	<0.001
Acceptance of the host culture	65.77 ± 17.92	65.77 ± 17.53	69.15 ± 18.08	66.31 ± 17.73	65.17 ± 17.98	<0.001
Psychological integration	79.07 ± 15.58	77.23 ± 14.61	81.43 ± 15.87	78.80 ± 15.50	79.36 ± 15.81	<0.001
**Mental health**						
Psychological distress	3.52 ± 3.09	3.25 ± 2.81	3.77 ± 3.40	3.49 ± 3.12	3.58 ± 3.13	<0.001
Perceived stress	5.39 ± 2.61	5.22 ± 2.58	5.37 ± 2.66	5.18 ± 2.82	5.46 ± 2.60	0.002

^a^Analysis of variance, rank-sum test or chi-square test.

M, mean; SD, standard deviation.

### Mediation effect of social integration on the relationship between migration paths and mental health

The results of multiple linear regression models (including social integration or not) were shown in Table 2. Compared with the new generation of rural-to-rural migrants, rural-to-urban migrants had higher scores of psychological distress (0.305, 95% CI: 0.152–0.458) and perceived stress (0.328, 95% CI: 0.199–0.456). When social integration was considered, the relationships between migration paths and psychological distress and perceived stress were weakened. Social integration generally improved the mental health of new-generation mi-grants, in which economic integration, acceptance of the host culture and psychological integration were negatively associated with both psychological distress and perceived stress scores.

**TABLE 2:** The relationship between migration paths, social integration and the mental health of new-generation migrants with multiple linear regression.

Variables	Psychological distress		Perceived stress	
	β (95% CI)	β (95% CI)	β (95% CI)	β (95% CI)
**Migration path**				
Urban-to-Urban	0.630 (−0.018,1.278)	0.624 (−0.002,1.250)	0.365 (−0.181,0.911)	0.410 (−0.117,0.932)
Urban-to-Rural	0.385 (−0.432,1.202)	0.312 (−0.475,1.099)	0.080 (−0.608,0.768)	0.044 (−0.612,0.701)
Rural-to-Urban	0.305 (0.152,0.458)**	0.235 (0.087,0.384)*	0.328 (0.199,0.456)**	0.286 (0.162,0.409)**
Rural-to-Rural	Ref.	Ref.	Ref.	Ref.
**Social integration**				
Economic integration	–	−0.044 (−0.050, −0.038)**	–	−0.045 (−0.050, −0.041)**
Life integration	–	0.008 (0.004, 0.012)**	–	−0.001 (−0.005, 0.003)
Maintenance of original culture	–	−0.001 (−0.005, 0.002)	–	−0.007 (−0.010, −0.004)**
Acceptance of the host culture	–	−0.024 (−0.027, −0.020)**	–	−0.021 (−0.024, −0.018)**
Psychological integration	–	−0.026 (−0.030, −0.022)**	–	−0.022 (−0.026, −0.019)**

*P < 0.05, **P < 0.001.

CI, confidence interval; Ref, reference group.

The results of bootstrapping test for mediating effect were shown in Tables 3, 4. It was found that two dimensions of social integration, namely life integration and acceptance of the host culture, mediated the relationship between the rural-to-urban migration path and the psychological distress of new-generation migrants by 0.022 (95% CI: 0.010–0.036) and 0.069 (95% CI: 0.047–0.094), respectively. Furthermore, in one dimension of social integration, the mediating effect of acceptance of the host culture on the relationship between the rural-to-urban migration path and perceived stress of new-generation migrants was 0.062 (95% CI: 0.043–0.082).

**TABLE 3:** The mediating effect of social integration on the association between migration paths and psychological distress of new-generation migrants.

Mediator	Migration paths (Ref = Rural-to-Rural)	Mediating effect	Bootstrap 95% CI	
			LL	UL
Economic integration	Urban-to-Urban	−0.078	−0.177	0.018
	Urban-to-Rural	−0.034	−0.166	0.096
	Rural-to-Urban	−0.014	−0.037	0.008
Life integration	Urban-to-Urban	0.057**	0.022	0.101
	Urban-to-Rural	0.014	−0.016	0.049
	Rural-to-Urban	0.022**	0.010	0.036
Maintenance of original culture	Urban-to-Urban	0.005	−0.008	0.021
	Urban-to-Rural	−0.002	−0.015	0.009
	Rural-to-Urban	0.001	−0.002	0.005
Acceptance of the host culture	Urban-to-Urban	−0.015	−0.106	0.076
	Urban-to-Rural	0.014	−0.098	0.126
	Rural-to-Urban	0.069**	0.048	0.093
Psychological integration	Urban-to-Urban	0.038	−0.039	0.113
	Urban-to-Rural	0.080	−0.021	0.187
	Rural-to-Urban	−0.009	−0.028	0.011

*P < 0.05, **P < 0.001.

CI, confidence interval; LL, lower limit; UL, upper limit; Ref, reference group.

**TABLE 4:** The mediating effect of social integration on the association between migration paths and perceived stress of new-generation migrants.

Mediator	Migration paths (Ref = Rural-to-Rural)	Mediating effect	Bootstrap 95% CI	
			LL	UL
Economic integration	Urban-to-Urban	−0.080	−0.180	0.015
	Urban-to-Rural	−0.035	−0.172	0.098
	Rural-to-Urban	−0.015	−0.038	0.008
Life integration	Urban-to-Urban	−0.007	−0.034	0.018
	Urban-to-Rural	−0.002	−0.014	0.007
	Rural-to-Urban	−0.003	−0.013	0.007
Maintenance of original culture	Urban-to-Urban	0.023**	0.002	0.050
	Urban-to-Rural	−0.009	−0.042	0.023
	Rural-to-Urban	0.005	−0.001	0.012
Acceptance of the host culture	Urban-to-Urban	−0.013	−0.097	0.065
	Urban-to-Rural	0.012	−0.090	0.115
	Rural-to-Urban	0.062**	0.043	0.082
Psychological integration	Urban-to-Urban	0.032	−0.032	0.095
	Urban-to-Rural	0.069	−0.018	0.154
	Rural-to-Urban	−0.007	−0.025	0.009

*P < 0.05, **P < 0.001.

CI, confidence interval; LL, lower limit; UL, upper limit; Ref, reference group.

## Discussion

Under the background of Chinese migrants, this study used the mediation analysis to explore the relationship between migration paths and the mental health of new-generation migrants through the five dimensions of social integration. The results showed that the mental health of the rural-to-urban migrants was worse than that of the rural-to-rural migrants, and social integration could improve the mental health of new-generation migrants. Furthermore, the relationship between the rural-to-urban migration path and the mental health of new-generation migrants was mediated by the two dimensions of social integration: life integration and acceptance of the host culture.

This study found that the new-generation migrants from rural to urban areas had worse mental health measured by psychological distress and perceived stress scores, which was consistent with evidence from previous studies (45, 46). The new-generation migrants tended to be more educated and more likely to develop in cities, but the income inequality caused by the registered permanent residence could lead to great stress and subsequent psychological distress (9, 47). Moreover, the differences between urban and rural areas would bring great difficulties to new-generation migrants when they came to the host city, which would affect their mental health (48). Unlike rural-to-urban migrants, urban-to-urban generation migrants were more likely to be the only child in a family, which means they would have more family expectations and responsibilities. Because of the deep-rooted belief in filial piety in Chinese society, Chinese only-child migrants experience a range of dilemmas, from personal development in a receptive society to caring for elderly parents (49). Similarly, urban-to-urban new-generation migrants were more motivated to pursue personal development in metropolitan areas than their rural-to-urban counterparts, and the longer they stayed in these areas, the less likely they were to return (19). The gap between expectations and reality could also lead to significant stress and subsequent mental health problems. The results of the study underscore the importance of providing mental health services to new-generation migrants.

Social integration was described by five dimensions (50, 51), and was found to improve the mental health of new-generation migrants, similar to previous research (52). During the process of migration, new-generation migrants would encounter various obstacles which may cause psychological pressure (53–55). However, the improvement of social integration might overcome these barriers, relieve these pressures and ultimately promote the mental health of new-generation migrants (56). In addition, resettlement-related stressors were important factors affecting the mental health of migrants, and strengthening social integration is the key to improving the mental health of new-generation migrants (57, 58).

This study observed that life integration and acceptance of host culture played a mediating role between the rural-to-urban migration path and the mental health of new-generation migrants. As a mediator, life integration weakened the negative effects of rural-to-urban migration on mental health. It may be that improved social insurance and active participation in society could help migrants obtain medical resources and health information, thus contributing to the mental health of new-generation migrants from rural to urban areas (59, 60). Acceptance of host culture as a mediator also weakened the negative impact of the rural-to-urban migration path on mental health. This was inconsistent with some previous studies, possibly because original and host cultures were not considered together in other studies (61). Cultural differences in migration paths could create psychological stress for rural-to-urban migrants (62). However, new-generation migrants from rural to urban areas were more likely to pursue an urbanized lifestyle, and greater acceptance of the host culture could alleviate these pressures and improve their mental health, as well as to urban-to-urban (63). Therefore, the government should attach great importance to the role of local culture and values for new-generation mi-grants and guide the local people to treat them with an open mind (64). Furthermore, the stressors after resettlement are the most important factors affecting the mental health of migrants. Targeting the sources of stress associated with resettlement through enhanced psychosocial care programs and social integration would be a key way to improve the mental health of migrants (58).

Some limitations of this study should be recognized. First, the Chinese version of migrants, which may not be fully applicable to reflect the real situation. But we calculated the Cronbach coefficients of K6 and PSS-4 were 0.83 and 0.61, respectively, which generally had good psychometric properties. Furthermore, due to the availability of data, migration paths were classified based on participants’ Hukou status using rural and urban areas rather than the level of economic development. And it was a limitation of our study that we could not meaningfully reflect the complexity of migration paths without considering the migration path from small towns to mega cities, so it is hard to comprehensively evaluate migration paths in a more detailed unit. However, most Chinese urban residents enjoy more advantages in income, education and employment than rural residents (65–67). Chinese medical resources are also concentrated in urban areas (68, 69). In 2020, for instance, the number of medical practitioners in urban China was 4.25 per 1,000 persons, compared with 2.56 per 1,000 persons in rural China (70). In addition, the rates of all-cause mortality and cancer mortality among rural residents were higher and increased faster than that of urban residents (71). Therefore, it could basically capture the economic-driven migration path trends in a rough way and this classification method has been verified in other studies (72, 73). Lastly, because this study was a cross-sectional design, it could not support the causal relationship between migration paths and the mental health of new-generation migrants, but only the correlation between them. Further longitudinal research may be required to verify in the future.

Despite these limitations, this study further added evidence of the effects of migration paths on the mental health of new-generation migrants and indicated the mediating role of social integration. In particular, the study relied on a large population sample covering a representative new-generation migrants in a developing country. The findings inform public policy makers that more migration-related policies are needed to promote the social integration of new-generation migrants and to make efforts to protect their mental health.

## Conclusion

This study highlighted that the rural-to-urban migration path had negative effects on the mental health of new-generation migrants. Furthermore, life integration and acceptance of the host culture as a mediator could weaken the impact. These findings suggested that migration policies should be developed to enhance the life integration and acceptance of the host culture and improve the mental health of new-generation migrants. At the same time, addressing the sources of stress associated with resettlement through enhanced psychosocial care programs and social integration would be a key way to improve the mental health of migrants.

## Data availability statement

The datasets presented in this study can be found in online repositories. The names of the repository/repositories and accession number(s) can be found below: https://chinaldrk.org.cn/wjw/#/application/index.

## Ethics statement

This study was a secondary analysis of a public access dataset of the National Internal Migrant Dynamic Monitoring Survey (NIMDMS) 2014. No identifiable private information of the participants was contained. This study was approved by the Ethics Committee of the School of Public Health, Sun Yat-sen University. The patients/participants provided their written informed consent to participate in this study.

## Author contributions

FZ, MC, BP, and LL: conceptualization. FZ, MC, and BP: data curation. FZ, MC, BP, and LS: formal analysis. FZ, MC, BP, LL, and HZ: funding acquisition. LL: resources. FZ and MC: writing—original draft preparation. FZ, MC, BP, HZ, LS, and LL: writing—review and editing. All authors have contributed to the data interpretation and have read and agreed to the published version of the manuscript.
