# Supplementary material for: Association between migration paths and mental health of new-generation migrants in China: The mediating effect of social integration
Source: Front Psychiatry. 2022 Sep 7;13:967291. doi: 10.3389/fpsyt.2022.967291 (PMC9490027; doi:10.3389/fpsyt.2022.967291)
Supplement: Supplementary file 1 [file Table_1.docx]

**Supplementary Table 1.** The social integration scale for the IMs in China in 2014.

| **Dimension** | **Factor** | **Item** | **Valuation** |
| --- | --- | --- | --- |
| **Economic integration** | Subjective socioeconomic status  (Cronbach's *α*=0.849) | 1. Where do you stand compared to your relatives, friends and colleagues back home? | Ladders represent people's status in society. If a person has the highest income and the best career, it is at 10. If someone has the lowest income and the worst career, it's at 1 |
|  |  | 1. Where do you stand compared to your relatives, friends and colleagues in your current place of residence? |  |
|  |  | 1. Where do you stand compared to the people in the whole society? |  |
|  | Objective socioeconomic status  (Cronbach's α=0.858) | 1. What is the total income of your family on average for each month? | The logarithm of the average monthly total income/expenditure in your family |
|  |  | 1. What is the total expenditure of your family on average for each month? |  |
| **Life integration** | Social insurance  (Cronbach's *α*=0.859) | 1. the number of the endowment insurance | 0 = None; > 1 or 1 = At least one |
|  |  | 1. the number of the medical insurance |  |
|  | Social participation  (Cronbach's *α*=0.557) | 1. the number of participation in social organizations | 0 = None; > 1 or 1 = At least one |
|  |  | 1. the number of participation in social activities |  |
| **Maintenance of hometown culture** | Maintenance of hometown culture  (Cronbach's *α*=0.800) | 1. It is important for me to abide by the customs of my hometown (such as the custom of weddings and funerals). | 1 = Strongly disagree; 2 = Disagree; 3 = Neither agree nor disagree; 4 = Agree; 5 = Strongly agree |
|  |  | 1. It is more important for me to follow the customs of my hometown. |  |
|  |  | 1. My child should learn to speak hometown |  |
|  |  | 1. Keeping hometown lifestyles (such as eating habits) is important to me |  |
| **Acceptance of local culture** | Acceptance of local culture  (Cronbach's *α*=0.872) | 1. My hygiene habits are quite different from those of local citizens. | 1 = Strongly agree; 2 = agree; 3 = Neither agree nor disagree; 4 = disagree; 5 = strongly disagree |
|  |  | 1. My dress is very different from the local citizens. |  |
|  |  | 1. My educational history or pension concept is quite different from that of local citizens. |  |
|  |  | 1. My views on some social issues are quite different from those of local citizens. |  |
| **Psychological integration** | the willingness to integration  (Cronbach's *α*=0.902) | 1. I would like to live with the locals in the same block(community) | 1 = Completely disagree; 2 = Disagree; 3 = Basic agree; 4 = Completely agree |
|  |  | 1. I would like to live next door to the locals |  |
|  |  | 1. I would like to work with the locals |  |
|  |  | 1. I would like to make friends with the locals |  |
|  |  | 1. I would like myself or a relative to marry a native |  |
|  | the sense of belonging  (Cronbach's *α*=0.886) | 1. I feel like I belong to this city | 1 = Completely disagree; 2 = Disagree; 3 = Basic agree; 4 = Completely agree |
|  |  | 1. I feel I am a member of this city |  |
|  |  | 1. I regard myself as a part of the city |  |
|  |  | 1. I would like to be part of the community |  |
|  |  | 1. I think the locals are willing to accept me as one of them |  |
